# Conservation of biodiversity in the genomics era

**DOI:** 10.1186/s13059-018-1520-3

**Published:** 2018-09-11

**Authors:** Megan A. Supple, Beth Shapiro

**Affiliations:** 1 Department of Ecology and Evolutionary Biology, University of California Santa Cruz, Santa Cruz, CA 95060 USA; 2 UCSC Genomics Institute, University of California Santa Cruz, Santa Cruz, CA 95060 USA

## Abstract

“Conservation genomics” encompasses the idea that genome-scale data will improve the capacity of resource managers to protect species. Although genetic approaches have long been used in conservation research, it has only recently become tractable to generate genome-wide data at a scale that is useful for conservation. In this Review, we discuss how genome-scale data can inform species delineation in the face of admixture, facilitate evolution through the identification of adaptive alleles, and enhance evolutionary rescue based on genomic patterns of inbreeding. As genomic approaches become more widely adopted in conservation, we expect that they will have a positive impact on management and policy decisions.

## Introduction

The human footprint on our planet is currently threatening biological diversity across habitats. Arguably the biggest threat to biodiversity across the planet is habitat degradation [1, 2]. As the human population increases, we modify the landscape to meet our increasing need for resources to support modern lifestyles. Coincident with this is an increase in energy consumption that is driving climate change across the globe. The rapid pace of the changing climate will outpace the natural ability of some species to respond [3, 4]. Temporal analysis of biodiversity loss indicates that we are on a trajectory for the Earth’s sixth mass extinction event [5], with the rate of extinction in the last century conservatively estimated to be 22 times faster than the historical baseline rate [6]. The picture is even more bleak when the analysis examines population declines, rather than the complete loss of species, with 32% of known vertebrate species showing substantial population declines [7].

Efforts to stop mass extinctions and population declines include setting up protected areas (for example, marine protected areas (MPAs)), international agreements to limit greenhouse gases to curb climate change (for example, the Kyoto Protocol and the Paris Agreement), and legal frameworks to protect endangered species (for example, the Convention on International Trade in Endangered Species of Wild Fauna and Flora (CITES) and the US Endangered Species Act (ESA)). Genomic technologies can aid these efforts by identifying biodiversity “hotspots” to prioritize for protection, using predictive models to help build natural communities that are resilient to environmental change, and informing management actions that attempt to mitigate threats to endangered species.

In this Review, we differentiate genetic approaches, which use a small number of neutral markers, from genomic approaches, which use complete genomes or genome-wide data. No standardized amount of data divides genetics from genomics; rather, this is a semantic distinction. We consider a study to have transitioned into the realm of genomics when a high density of markers is assayed from across the entire genome, usually in the order of thousands of markers.

Although both genetic and genomic data sets can be used to estimate genetic diversity, population structure, and demographic history, genome-scale data, with an increased density of markers across the genome, can provide more accurate estimations of these parameters [8–12], sometimes resulting in different conservation recommendations. For example, an analysis of more than 25,000 loci in the foothill yellow-legged frog revealed strong differentiation between five phylogenetic clades that the researchers suggested should provide the foundation for the management of the species; whereas a previous analysis of 1,525 bp of mitochondrial DNA (mtDNA) did not have the resolution to recover these clades and instead recommended conservation based on hydrologic boundaries [13]. Similarly, an analysis of 3,095 single nucleotide polymorphisms (SNPs) in the eastern tiger salamander found that roads restricted movement between ponds; however, a prior study using [12] microsatellite loci to examine the same ponds found high migration rates between ponds [14]. The most recent study suggested that mitigation of the impact of roads on the connectivity between ponds would be an important conservation target [14].

In addition to the increased precision of estimates of traditional parameters, the transition to genomic approaches allows researchers to ask qualitatively different questions. This is because our capacity to examine different evolutionary mechanisms increases with the amount of the genome interrogated. In addition to assaying putatively neutral loci and protein-coding regions of the genome, whole-genome sequencing allows the identification of non-coding regulatory regions that control gene expression, and whole-transcriptome sequencing allows the quantification of gene expression differences.

The limited use of genome-scale data in a conservation context is probably due to the additional challenges presented by these data sets. One important consideration is cost. Although the cost of sequencing continues to decrease, most conservation projects have limited budgets that allow genome-scale sequencing of only a small number of samples. The tradeoff between the number of samples and the number of loci sequenced is a critical consideration, and the best approach in each case will depend on the specific research question. Another important consideration is data analysis; that is, the specific resources and expertise that are available to analyze whole-genome data. Calling genotypes requires a reference genome, which may not be available for many non-model organisms, and analysis software is not always user-friendly. Finally, once a researcher obtains results from whole-genome analyses, it is often difficult to interpret the results and to translate them into conservation recommendations.

In this Review, we discuss how conservation researchers and managers can use the power of genomic data to make decisions on the conservation of biodiversity. We focus on conservation topics where genome-scale data can provide valuable insights that are unattainable with traditional genetic techniques: delineating species in the face of admixture, identifying adaptive alleles through association mapping, and enhancing evolutionary rescue based on genomic patterns of inbreeding.

## Admixture and species delineation

The current conservation regulatory framework relies on defining distinct units of conservation to support law enforcement and to inform resource allocation. In conservation, the term “species” is often used to convey the idea of a unit of conservation and includes taxonomic levels below species, such as subspecies and distinct populations. Defining specific species is fraught with challenges and differing opinions [15]. In conservation, researchers tend to prefer a phylogenetic species concept, which identifies species based on their apparent differences [16–18], but which may over-split groups [19]. Other common species concepts require estimations of genetic distances or proof of reproductive isolation, which are challenging data to gather from most natural populations. Disagreement over how species should be defined highlights both the artificial nature of species as purely discrete units and the importance of defining species in biology, where a means to categorize organisms provides a framework for hypothesis testing. Several discussions of the relevance to conservation of defining species have recently been published, and we refer the interested reader to these [16, 18, 19].

Identifying and describing conservation units is often confounded by the lack of clear boundaries between management units. The most common categorization currently in use is the evolutionarily significant unit (ESU), which defines a group as distinct if it is “substantially reproductively isolated from other conspecific population units” and “represents an important component in the evolutionary legacy of the species” [20]. An ESU of a vertebrate species can be defined as a distinct population segment (DPS), which is the smallest biological categorization that can be listed under the ESA. ESUs and DPSs are populations that may be geographically isolated or that may be morphologically distinct from other populations of the same species, and may also be distinct based on some measure of genetic divergence [20–22]. There is no strict rule, however, regarding the amount of genetic divergence required to qualify as an ESU or a DPS; definitions of genetic distinctiveness for ESUs range from significant divergences in allele frequencies to a consistent phylogenetic signal across multiple tested genes [21, 22].

**Fig. 1:**
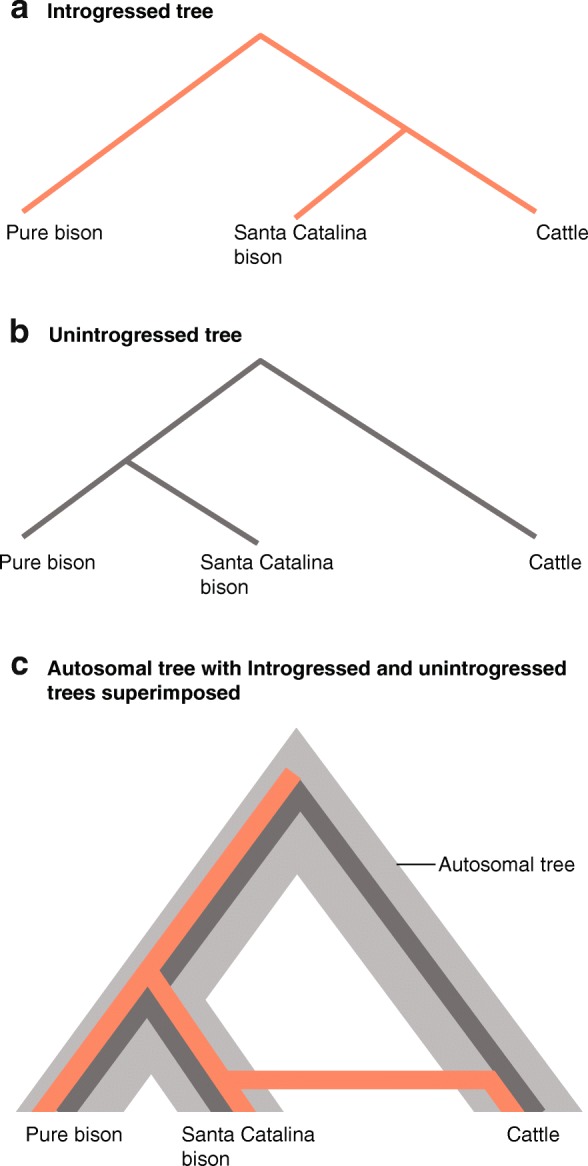
Variation in evolutionary history due to admixture, using American bison as an example. **a**, **b** The two different evolutionary histories that are present in the genomes of bison from the Santa Catalina Island herd. **c** The autosomal tree (*gray*) with the two different mitochondrial trees superimposed (*red* and *black*). Examining autosomal markers, 99.4% of the population is represented by the unintrogressed tree. Examining mitochondrial markers, 55.1% of the population is represented by the unintrogressed tree (*black*), and 44.9% follow the introgressed tree (*red*). Data from Hedrick [23]

In evolutionarily simple biological systems, traditional genetic techniques can delineate conservation units in a straightforward manner. However, the task is more complicated in complex evolutionary systems, such as those with a history that includes admixture and introgression [17]. Admixture is the interbreeding between individuals from distinct groups, such as that between two related species. Introgression is the transfer of alleles from one species to another. Admixture and introgression complicate the task of delineating units of conservation because analyses of different parts of the genome can result in qualitatively different answers. This conflict can be seen in the genomes of plains bison (Fig. 1), which have a known history of admixture with cattle. An analysis of the mtDNA of a Santa Catalina herd indicated that the herd’s ancestry is 44.9% cattle; but an analysis of the autosomal DNA indicated that the herd’s ancestry is only 0.6% cattle [23].

Genomic research has revealed a high frequency of admixture in natural systems, ranging from great apes to bears and butterflies [24–26]. For example, evidence of admixture between ancient anatomically modern humans and archaic hominins is written into the genomes of most present-day humans, who individually contain up to 7.4% ancestry from Neanderthals and Denisovans [27, 28]. As genome technologies and genomic resources have improved, so have the statistical methods to detect and quantify admixture. It is now possible not only to detect ancient admixture, but also to examine the genomic signatures of admixture on a fine scale. Researchers are now able to detect rare admixture events; however, these rare events may not be critical components of the evolutionary history of the species, so their identification may cloud attempts to delineate units for conservation. More importantly, high-resolution genomic data enable researchers to infer ancestry for specific regions of the genome and to estimate the timing of admixture events [29–31].

Understanding the admixture histories of natural populations is important when delineating units for conservation, as admixture erodes the genetic distinctiveness on which conservation units are based. Historically, this has led admixture to be seen in conservation as a threat to the integrity of endangered species [32–35]. More recently, as genomic research has revealed its commonness in evolutionary history, admixture has come to be viewed as a potential source of new genetic variation [32–34, 36–38]. In this view, the influx of new genes from admixture can be seen to provide critical variation on which natural selection can act. This new variation may be vital, for example, in highly inbred populations or in populations at the edges of their ideal habitat range where rapidly changing environments may pose a considerable threat.

Given that conservation legislation is based on the identification of distinct units, it is not surprising that regulations also vary with respect to how hybrid populations should be protected [32, 34]. Some conservation policies favor the eradication of admixed populations, particularly if admixture has occurred because of human intervention [39]. Even policies that do not favor eradication tend to provide few specific guidelines for categorizing admixed populations [32]. This practice leads to policy implementation that varies from no protection to complete protection for admixed individuals [32].

Although genomics will not solve the problem of discrete classification in an inherently non-discrete system, genome-scale data can provide researchers and managers with a more complete understanding of the spatial and temporal dynamics of admixture in evolutionarily complex systems. Much research in this realm has taken place in naturally occurring hybrid zones where one of the two parent species is protected. In both genetic and genomic approaches, the main goal is to identify ancestry-informative markers that are capable of distinguishing the two parent-species and estimating the proportion of ancestry of the protected parent species in hybrid individuals. For example, using a genetic approach, researchers used amplified fragment length polymorphisms (AFLPs) to determine parentage in hybrid garter snakes in Wisconsin [40]. Despite the limitations of AFLPs as genetic markers, this research provided important insights to managers. The AFLPs proved that nearly genetically pure members of the protected species occurred throughout the garter snake hybrid zone [40]. This finding suggests that unless morphologically diagnostic characteristics are identified, protection of the endangered garter snake would only succeed if both species were protected in regions where their ranges overlapped.

As DNA-sequencing technologies advance, so does the ability to sequence markers more densely across the genome, which both improves parentage estimates and provides a means to identify patterns of genetic introgression, with potential conservation implications. For example, researchers used expressed sequence tags (ESTs) to identify SNPs that were fixed for different alleles between the threatened California tiger salamander and the intentionally introduced non-native barred tiger salamander [41]. Researchers identified 68 ancestry-informative SNPs and used these SNPs to quantify ancestry. They then tracked the spread of these invasive alleles by mapping marker allele frequency against geographic distance from a known introduction site. Although 65 invasive alleles did not spread far from the introduction sites, the remaining three have moved 90 km in the 60 years since the introductions began, indicating that alleles can move at different rates across the landscape. Additional insights into the implications of hybridization may be obtained through whole-genome sequencing, which takes a more complete look at the genome than do ESTs by allowing the interrogation of unexpressed regions of the genome, such as non-coding regulatory sequences. However, for these salamander species whole-genome sequencing is currently impractical, as both species have genomes in excess of 30 Gb. Exome-capture methods are in development to provide high-density genome-wide markers with the aim of addressing these questions [42]. From a conservation perspective, this research highlights how difficult it might be to contain invasive alleles once they are introduced, suggesting that a goal of maintaining “pure” species in the face of hybridization may be impractical [41].

Genomic inference can, however, help to protect specific traits. For example, by combining fine-scale genomic data with phenotypic data, it is possible to connect particular genomic regions to ecologically important traits (as discussed below in the “Association mapping and adaptation” section). This would provide an improved understanding of the ecological consequences of introgression and may lead to targeted efforts to protect individuals that carry these traits. The ability to identify adaptive genetic variation raises the possibility of using this variation to delineate conservation units. For example, a proposal has been submitted to list spring-run Chinook in the Klamath River as endangered under the ESA based on a single allele that is strongly associated with the spring-run phenotype [43–45]. This proposal, however, has been controversial, with some parties highlighting the need to protect adaptive variation, and other parties more concerned about the implications of conservation decisions based on single-gene analyses [43]. Others argue that, rather than focusing on a few genes and traits of interest, delineation of conservation units should include genome-wide signatures of adaptation [21]. Important to this ongoing debate is to acknowledge that methods used to identify genome-wide adaptive variation are hampered by high rates of false negatives and false positives [46, 47]. The outcome of this specific debate is as-yet unknown, but will no doubt have implications for conservation genomics research and practice.

**Fig. 2:**
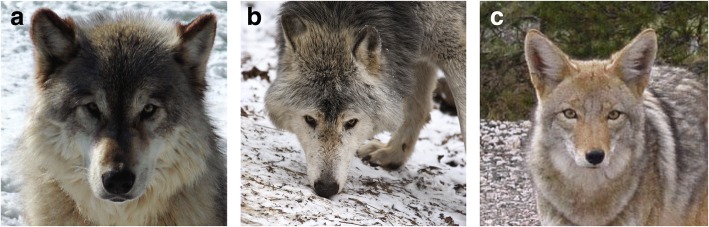
Photos of a (**a**) gray wolf (photo by Derek Bakken), (**b**) an eastern wolf (photo by Christian Mehlführer), and (**c**) a coyote. Photos from Wikimedia Commons

Another notable example of using genome-scale data to delineate conservation units is in the wild canids of North America. Whole-genome sequencing was used to detect admixture and to disentangle the complex evolutionary history of wolves and coyotes (Box 1). Gray wolves in North America have been divided into several subspecies: *Canis lupus baileyi*, *Canis lupus nubilus*, *Canis lupus occidentalis*, *Canis lupus arctos*, and *Canis lupus lycaon* (the eastern wolf) (Fig. 2) [48]. The taxonomic status of the eastern wolf has been controversial in large part due to a complex history of admixture with coyotes. This has implications for conservation because the eastern wolf is currently protected as a subspecies of gray wolf. However, the US Fish and Wildlife Service (USFWS) has suggested that the eastern wolf is instead a longstanding lineage native to eastern North America that was derived from a common ancestor with the coyote and has recently admixed with gray wolves. Given this taxonomic revision, the eastern wolf is not protected under the gray wolf ESA listing [49, 50]. Using a high-density domestic dog SNP chip and whole-genome sequences, researchers found that qualitative patterns of variation across the genome indicate that the eastern wolf is of gray wolf ancestry with recent admixture with coyotes [51, 52]. They estimated the time since admixture using SABER software, which models ancestry blocks using a Markov-hidden Markov model (MHMM) and accounts for ancestral linkage disequilibrium [53]. They estimated that admixture occurred approximately 600–900 years ago, which is prior to the invasion of coyotes into areas occupied by the eastern wolf [51]. They inferred that admixture with coyotes may have been an important component in eastern wolf evolution [51]. However, SABER does not model haplotype structure, which provides additional information on the timing of admixture events [54]. Additionally, programs such as SABER only estimate the time since admixture when there has been a single admixture event [53]. The development of new statistical methods that can disentangle multiple admixture events that occur across the evolutionary history of a species will be informative for this and other conservation decisions relating to admixed species.

### Box 1: Conservation implications of admixture in the eastern wolf

The history of federal protection of the gray wolf in the US spans 50 years. Initially, individual gray wolf subspecies were protected separately. In 1978, the US Fish and Wildlife Service (USFWS) issued a ruling under the US Endangered Species Act (ESA) to reclassify the gray wolf as endangered at the species level, protecting gray wolves throughout the lower 48 states and Mexico. Some gray wolf populations have since recovered and six states have been removed from the 1978 listing. Gray wolves are currently protected in 42 states and Mexico [49]. In 2013, the USFWS proposed delisting the gray wolf based on a taxonomic revision by USFWS scientists [49, 50]. The revised taxonomy considers the eastern wolf subspecies, *C. lupus lycaon*, a separate species, *C. lycaon*, and means that the current listing for *C. lupus* is invalid as it includes 29 states that are occupied by *C. lycaon* rather than by *C. lupus* [49]. In addition, in reassessing the status of *C. lupus* based on the new taxonomy, the USFWS found that *C. lupus* was neither threatened nor endangered, with the exception of the subspecies *C. l. baileyi* in the southwestern US and Mexico [49].

**Fig. 3:**
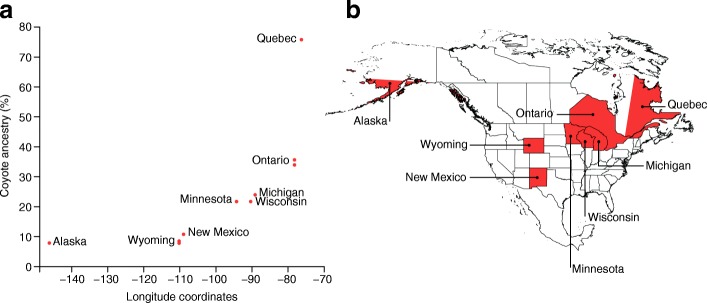
**a** Geographic cline of coyote ancestry in gray wolves. Coyote ancestry increases towards the eastern portion of the range, coincident with the increased persecution of wolves that reduced population densities, resulting in an increased probability of admixture with other canid species. Samples are labeled with the geographic location of collection. **b** The state or province of the sample collection (*red*). Data from vonHoldt et al. [52]

The 2013 taxonomic revision that led to the proposed delisting of the gray wolf has proved to be as controversial as other aspects of wolf protection and recovery in the US. Although there seems to be agreement that admixture is an important component in explaining patterns of genetic variation in eastern wolves, there is disagreement about the context of admixture and the implications for canid taxonomy. Chambers et al. (2012) argue that, based on a review of the existing literature, the eastern wolf evolved in North America from a common ancestor with coyotes and now hybridizes with gray wolves where their ranges overlap [50]. They cite phenotypic differences and concordant uniparentally inherited markers (Y chromosome and mitochondrial DNA (mtDNA)) as supporting a species-level distinction. Additionally, they note that geographic discontinuities in microsatellite data also indicate isolation and are consistent with spatially associated admixture. vonHoldt et al. (2011, 2016), using a high-density domestic dog SNP chip and whole-genome sequences, argue that the eastern wolf is instead a more recent lineage and is derived from the gray wolf [51, 52]. Their results indicate recent admixture with coyotes and show a geographic cline in the amount of coyote ancestry (Fig. 3). This cline can be explained by spatial patterns of wolf persecution by humans that result in lower population densities, decreasing the probability of finding a conspecific mate and thereby increasing the probability of admixture with other canid species [52]. They argue that this recent admixture could be driving the phenotypic differences that are the primary evidence of the species designation by Chambers et al. [51].

Determination of the historical context of admixture has implications for conservation. An admixed species is viewed differently if admixture is a natural part of its evolutionary history versus for a species where admixture is recent and driven by human activities [39, 55]. Formal model testing should be used to test specific hypotheses, as the data may be consistent with different hypotheses, each of which could have diverse implications for conservation and management. Additionally, haplotype analysis with whole-genome data will be necessary to estimate the timing of admixture events and new statistical approaches are needed to determine whether ancient hybridization, in addition to more recent admixture, was an important component of the evolutionary history of eastern wolves.

## Association mapping and adaptation

Adaptation is a genetic process that allows a species to persist for generations in a changing habitat. A central focus of traditional conservation genetics has been to ensure that populations maintain sufficient genetic variation to act as substrates for the process of adaptation. With the transition to modern high-resolution genomic data, conservation researchers can not only assay overall levels of genetic variation, but also identify specific alleles that may be adaptive. Such data can provide managers with useful information when they need to prioritize populations for protection or need to make decisions regarding which individuals to translocate so as to boost diversity in a declining population.

Candidate loci underlying a particular phenotype can be determined through association mapping, which searches for an association between genotype and phenotype. Genome-wide association studies (GWASs) are commonly used to delineate the genetic basis of diseases in humans [56, 57]. Using a case–control design, researchers identify genetic variants that are highly correlated with disease status in individuals. Although identifying causative mutations requires follow-up studies, the correlation between genotype and phenotype enables the estimation of an individual’s risk of developing disease, given the individual’s genotype [58].

**Fig. 4:**
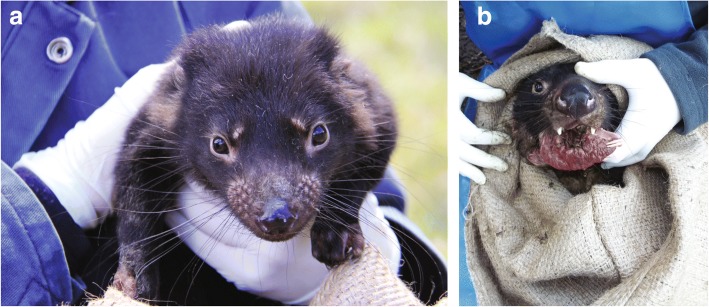
Photos of a healthy (**a**) and a diseased (**b**) Tasmanian devil. Photos courtesy of the Save the Tasmanian Devil Program

**Fig. 5:**
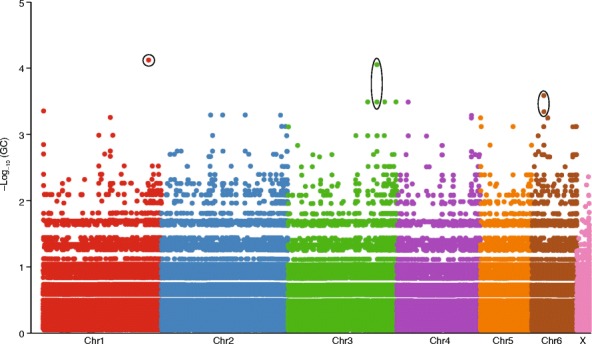
Manhattan plot of adjusted *p* values of the Tasmanian devil genome-wide association study (GWAS) comparing seven devils that recovered after infection with devil facial tumor disease (DFTD) to six devils that succumbed to the disease. Scaffolds are placed on chromosomes but are unordered. *Circles* indicate the five candidate SNPs, which are located on three scaffolds. Four of the candidate SNPs (on Chr3 and Chr6) remained significant after including additional samples. Data courtesy of Wright et al. [61]

Association mapping can be useful in conservation when an identifiable phenotype has clear fitness consequences in the population of interest. Disease resistance is a particularly important target that may have implications for management decisions. For example, the Tasmanian devil is at risk of extinction due to devil facial tumor disease (DFTD) [59] (Box 2, Fig. 4). DFTD is almost always fatal [60]; however, in a single population, a small number of infected devils have naturally recovered from the disease [61]. If this resistance to DFTD has a genetic basis, the identification of the specific genetic variants underlying resistance could be informative for conservation [59]. Using a GWAS to compare whole-genome sequences for seven devils that recovered from the disease with those from six devils that succumbed to the disease, researchers identified three regions where genotype was associated with disease status (Fig. 5) [61]. To validate candidate loci, the researchers performed targeted genotyping of five SNPs from the three genomic regions in a single additional recovered devil and 13 additional susceptible devils. Association analysis of genotypes from all 27 devils confirmed the association between four of the SNPs in two of the genomic regions [61].

This study highlights one of the challenges of using GWASs in endangered species—the very small sample sizes and the often close relatedness of individuals in the data sets. These characteristics reduce the power of GWASs and can lead to spurious results [62]. Although extensive experimental work would be required to determine whether variants in the identified regions are the cause of resistance to DFTD, even the suggestion of an association is important to consider when making management decisions. For instance, these genetic variants should be included in the captive breeding programs that have been developed to ensure a disease-free insurance population for reintroduction. Additionally, individuals carrying variants that might provide resistance to DFTD can be considered for translocation to other populations [59]. It also calls into question the practice of culling diseased animals (which was found to be ineffective in trials [63, 64]) because it could remove natural genetic variation that provides disease resistance.

A similar process can be applied to identifying adaptive genetic variation that reduces the susceptibility of coral species to bleaching, which can be used to increase the resilience of reef ecosystems to warming oceans. Corals are the foundation species of the reef ecosystem, so coral bleaching is a major threat to the entire ecosystem [65]. Coral bleaching is a stress response to high ocean temperatures that disrupts the symbiotic relationship between corals and algae [66]. Researchers have identified alleles that potentially confer a tolerance to bleaching using a natural temperature mosaic across a small area where corals that are located in higher temperature microclimates were found to be more resistant to bleaching [67]. Using cDNA sequencing, researchers identified 114 SNPs that showed a strong association with the local temperature regime [67]. The researchers then measured the allele frequencies of these adaptive alleles in another population and incorporated that information into models of evolutionary adaptation to predict whether corals will survive under various climate change scenarios [68]. Under optimistic climate change models, the presence of thermal-tolerant alleles at the low frequencies measured in the additional population, which currently experiences cooler microclimates, was sufficient for natural adaptation to increasing temperatures. However, under pessimistic climate change predictions, adaptation was too slow and species extinction was predicted unless a substantial transplantation effort was undertaken to increase the rate of adaptation [68].

### Box 2: Tasmanian devil facial tumor disease

The Tasmanian devil is one of the iconic animals of Australia, but this carnivorous marsupial is at risk of extinction due to devil facial tumor disease (DFTD) [59]. DFTD was first observed in 1996, when the species was considered to be healthy, with an International Union for Conservation of Nature (IUCN) status of “lower risk/least concern”. In the 10 years after the emergence of DFTD, the species declined by more than 60% and was then listed by the IUCN as “endangered” [59]. DFTD is a transmissible cancer that spreads between devils when they bite each other during feeding and mating [69]. DFTD has an extremely high mortality rate [60], with death usually resulting from organ failure that is associated with metastases or starvation when the tumors interfere with an individual’s ability to feed [70].

Genomics research on both tumor and host genomes has greatly increased the understanding of the disease and has informed potential management actions. Comparison of host and tumor karyotypes found complex rearrangements to be present in all tumor samples but absent from the host samples, indicating that the tumors were all derived from the same source [69]. In further support of this hypothesis, a single host devil had an inversion in its genome that was not present in its tumor, indicating that the tumor was not derived from the devil’s own tissue [69]. The clonality of DFTD was confirmed by comparisons of tumor and host microsatellite genotypes, mitochondrial sequencing, and microRNA expression, all of which cluster tumors separately from hosts [71]. Differential expression analysis of tumor and non-tumor host tissue identified Schwann cells as the likely origin of the cancer cell line and provided a diagnostic test using tumor staining with an antibody against periaxin (PRX), which is a Schwann cell-specific myelin protein [71].

The discovery that DFTD is a clonally transmissible cancer led to research to determine why the devils failed to reject the foreign cell line. Researchers examined the major histocompatibility complex (MHC), which plays an important role in the recognition of foreign molecules by the immune system. Sequencing the host MHC revealed low diversity, which was consistent with reduced immune function [72]; however, consistent rejection of experimental skin grafts indicated that the devils’ immune systems were functional [73]. DFTD instead seems to evade the host immune system by altering gene expression to prevent the expression of cell surface MHC molecules [74]. This research has led to a potential vaccine and treatment using DFTD cells that express surface MHC molecules. This protocol has been shown to be effective in a small study [75]. Other potential vaccine or treatment targets were identified using a genome-wide association study (GWAS) in a small number of devils that showed spontaneous recovery from DFTD [61] (see main text). This analysis identified two genomic regions where genotypes were strongly associated with disease survival. Both genomic regions are associated with angiogenesis (new blood vessel formation), and further investigation may clarify the mechanism that allows devils to recover from this usually fatal disease [61].

## Genomic inbreeding and genetic rescue

Genetic rescue is a conservation tool used to increase the fitness of at-risk populations by introducing new genetic variation into the population. This is usually accomplished by translocating individuals from a closely related population (assuming that such a population exists) into an at-risk population. Genetic rescue is expected to be most useful for small, isolated populations that suffer from inbreeding [76]. Theoretical models suggest that such populations have lower fitness because they carry an increased genetic load: the reduced efficiency of selection and the increased action of drift is predicted to allow mildly deleterious alleles to drift to high frequency [77]. The goal of genetic rescue is to introduce new genetic variants that contain more favorable alleles, thereby reducing the genetic load [78]. This potentially powerful conservation tool is rarely used, in part because of concerns over outbreeding depression and the difficulty in predicting the outcomes of planned genetic rescue programs [76, 79].

To make decisions regarding genetic rescue, it is important to understand the level of inbreeding in the population, which depends on the size of the population and its demographic history. A common way to estimate the level of inbreeding is to calculate a genome-wide estimate using either genetic markers or a pedigree. Because genetic markers estimate realized inbreeding but pedigrees estimate expected inbreeding (including a large variation due to stochastic processes), estimates from genetic markers are more accurate [80–82]. Additionally, pedigrees often lack sufficient depth to capture inbreeding events that occurred more than a few generations previously [81–83]. Recently, whole-genome sequencing has enabled a transition from focusing on genome-wide estimates of inbreeding to examining patterns of inbreeding across the genome. Homozygous genomic regions within an individual, which are a result of inbreeding, are broken down over time by recombination. Therefore, the lengths of runs of homozygosity can be used to estimate the timing of inbreeding events [82, 84].

In addition to estimating the timing and the level of inbreeding, it is useful to estimate the deleterious fitness effects that result from an increase in homozygosity. The fitness effects of a particular genetic variant in a protein-coding sequence can be predicted from models of protein structure and by comparing the level of sequence conservation across species [85, 86]. Predicting the fitness effects of these variants across the genome allows the estimation of the genomic load of deleterious alleles carried by a population [85]. Combining this information with patterns of inbreeding across the genome can identify candidate loci underlying inbreeding depression, as predicted deleterious alleles that occur in homozygous regions may be causing phenotypic defects [82]. Conversely, regions of consistently high heterozygosity in otherwise homozygous genomes may be harboring recessive lethal alleles, with individuals not surviving if they are homozygous for the deleterious allele.

Genomic information about inbreeding and deleterious alleles can be valuable for managers who are considering a genetic rescue program. First, researchers should determine whether the population has reduced genetic diversity and a substantial amount of inbreeding. If so, they should then determine whether the inbreeding is predicted to have negative consequences on the fitness of the population and whether genetic rescue is predicted to increase fitness. If managers decide to establish a genetic rescue program, they should then decide which populations and which individuals will be used as the source of translocations into the at-risk population. All of these decisions, including the decision not to initiate a rescue program, rely on being able to predict the genomic consequences of the different available options.

One classic example of a successful genetic rescue is the Florida panther, a subspecies of mountain lion [87]. By the early 1990s, Florida panthers were critically endangered, with only 20–25 adult panthers living in the wild. Severely reduced genetic variation and high levels of inbreeding were causing phenotypic defects, including poor sperm quality and cardiac abnormalities. Given the high likelihood of extinction, the decision was made to translocate eight wild mountain lions from the Texas subspecies, reopening historical gene flow between these two populations. As a result of the genetic rescue combined with other management actions phenotypic defects decreased and the population size increased [87].

Genetic rescue, however, is not always successful, as seen with the wolves of Isle Royale National Park [88]. It was hoped that a natural migration of a single wolf in 1997 might genetically rescue this small and isolated island population. Initially, the influx of new genetic material seemed to increase the fitness of the wolf population. But as the migrant’s genotype swept to high frequency, the population began to decline, until a population low of two highly related adult wolves in 2016 [88, 89]. Researchers hypothesize that the migrant carried recessive deleterious alleles, the nature of which were masked by heterozygosity in the early generations but were revealed with increasing homozygosity in subsequent generations [88, 89]. Genomic analyses, particularly the identification of deleterious alleles, may have been able to predict the failure of this genetic rescue. The USFWS has approved a plan to reintroduce 20–30 wolves to Isle Royale over a 3-year period [90, 91], so the ability to predict the genomic consequences of reintroductions may help to select individuals that will support a healthy population. However, in most non-model systems with limited genomic resources, the accuracy of predictions of the fitness effects of particular genotypes are similarly limited.

Even in systems that are able to leverage the genomic resources of model systems, it has proved difficult to connect predicted high deleterious loads to decreased population fitness. The Channel Island fox, for example, occurs in only very small and isolated populations. Genomic analyses using genomic resources developed for domestic dogs revealed extremely low levels of genomic diversity and an increased load of deleterious mutations in Channel Island fox populations [92]. These genome characteristics suggest that the populations should have low fitness and should be at risk of extinction; however, Channel Island fox populations seem to be healthy, perhaps because of their ecologically stable and low-stress environment in which they lack competitors and predators [92]. Another species in which deleterious mutational load has been estimated is the critically endangered mountain gorilla, which shows similar genomic patterns of low genome-wide diversity, long runs of homozygosity, and a predicted high load of deleterious alleles [93]. It is unclear whether the decline in genetic diversity in mountain gorillas is causing a decline in fitness, but researchers have observed phenotypic signs of inbreeding [93].

Increasing genomic resources in model and non-model systems, combined with improved prediction algorithms, should help researchers and managers to better identify at-risk populations and to understand the genomic and fitness consequences of different proposed management actions.

## Future prospects

Genomic sequencing is helping to inform conservation decisions by providing critical information regarding species of conservation concern. Although the current focus of conservation genomics is on monitoring and managing existing genomes of species, new genomic technologies will allow researchers to manipulate genomes to help achieve conservation goals. Genome-editing technologies such as CRISPR–Cas9 [94, 95] allow precise genome editing at relatively low cost. Using a guide RNA to identify a specific region of the genome, the CRISPR complex binds to the target DNA and cleaves it. The DNA repair mechanism fixes the double-stranded DNA break, resulting in a sequence modification that is likely to knock out the function of the gene. Alternatively, a template sequence can be added to the CRISPR complex and used for repair, allowing the insertion of a specific sequence with desired genome edits [95].

The ability to use genome editing to replace alleles might enable researchers to assist the evolution of species by improving disease resistance or by enhancing adaptation to changing climates. For example, an older gene transfer technology, *Agrobacterium-*mediated transformation, has been used to incorporate fungal blight-resistant genes from wheat into the American chestnut tree, which is nearly extinct as a result of an introduced fungal pathogen [96]. This modified strain is being outcrossed with the existing American chestnut gene pool via natural stump sprouts that remain after the trees have succumbed to the blight. This method incorporates blight resistance into the existing genome-wide diversity with the hope of producing blight-resistant American chestnut trees in their native range [96].

A similar plan has been proposed to save the critically endangered black-footed ferret. The black-footed ferret was once widespread across the Great Plains, but a combination of factors, including habitat loss and disease, caused its extinction in the wild [97]. A successful captive breeding program was initiated, but reintroduction has been hampered by the susceptibility of black-footed ferrets to sylvatic plague [98]. A proposal has been submitted to the USFWS to use genetic engineering to induce plague immunity in captive-bred black-footed ferrets using DNA sequences from plague immunity alleles from the domestic ferret [99, 100].

Similarly, genetic-engineering techniques could be applied to help corals become more resistant to rising water temperatures. Heat-resistant alleles could be engineered from heat-tolerant corals and introduced into susceptible corals. To this end, work has already begun to develop CRISPR techniques in coral symbionts to increase the resilience of coral reefs to climate change-related stressors [101].

Other applications of genome-editing technologies with potential use in conservation are gene drives. Genetically engineered gene drives increase the inheritance of the engineered allele to spread the desired trait through the population [102, 103]. Gene drives are currently being tested in mosquitos with the goal of controlling malaria in human populations [104], but this technology could be transferred to control avian malaria, which has been introduced to Hawaii and is a major cause of bird population declines [105]. Gene drives are also a promising method of eradicating invasive rodents from islands by using methods to alter sex determination, resulting in reduced reproduction until the invasive species is extirpated from the island [105].

New technologies also allow us to move beyond making small changes to the genome. For instance, cloning by somatic cell nuclear transfer has been proposed as an approach to reintroduce lost genetic material into the black-footed ferret using preserved cell lines from an extinct lineage [106]. In the future, genomic technologies may even allow us to resurrect important ecological traits that disappeared when species became extinct [107], potentially redressing past effects that humans have had on ecosystems.

Whether using traditional conservation genetics or cutting-edge genomic engineering, any action taken—or not taken—comes with practical, legal, and ethical issues that need to be discussed with researchers, managers, and the public [103, 105, 108]. With emerging technologies in mind, seed banks and frozen zoos can ensure that existing genetic variation is preserved [109, 110]. These archives are not intended to replace traditional conservation measures, but rather they should act as insurance policies. Genetic material that is saved now may be able to be used for the currently unimaginable genomic technologies of the future.

## Conclusions

As human activities drive our planet into its sixth mass extinction event, genomic technologies will be an important tool for conservation researchers, helping to provide valuable scientific information to managers and policy makers. Genetic approaches have a long history of use in conservation, but the transition to genomic technologies is only just beginning. By expanding available data sets to the genomic scale, researchers can ask and answer different questions and can thus gain valuable insights that will be applicable to conservation. As genomic technologies continue to advance, the potential for these technologies to impact conservation decisions increases. The knowledge we gain will hopefully enable us to mitigate our impact on the earth’s biota.

## Abbreviations

AFLP: Amplified fragment length polymorphism
DFTD: Devil facial tumor disease
DPS: Distinct population segment
ESA: Endangered Species Act
EST: Expressed sequence tag
ESU: Evolutionarily significant unit
GWAS: Genome-wide association study
IUCN: International Union for Conservation of Nature
MHC: Major histocompatibility complex
USFWS: US Fish and Wildlife Service
